# Vortioxetine hydrobromide inhibits the growth of gastric cancer cells in vivo and in vitro by targeting JAK2 and SRC

**DOI:** 10.1038/s41389-023-00472-4

**Published:** 2023-05-05

**Authors:** Mingzhu Li, Lina Duan, Wenjie Wu, Wenjing Li, Lili Zhao, Ang Li, Xuebo Lu, Xinyu He, Zigang Dong, Kangdong Liu, Yanan Jiang

**Affiliations:** 1 Department of Pathophysiology, School of Basic Medical Sciences, Academy of Medical Science, Zhengzhou University, Zhengzhou, 450000 China; 2 China-US (Henan) Hormel Cancer Institute, Zhengzhou, 450000 Henan China; 3 State Key Laboratory of Esophageal Cancer Prevention and Treatment, Zhengzhou, 450000 Henan China; 4 Provincial Cooperative Innovation Center for Cancer Chemoprevention, Zhengzhou University, Zhengzhou, 450000 Henan China; 5 Cancer Chemoprevention International Collaboration Laboratory, Zhengzhou, 450000 Henan China; 6 Center for Basic Medical Research, Zhengzhou University, Zhengzhou, 450000 Henan China

**Keywords:** Cancer prevention, Oesophageal cancer, Target identification, Target validation

## Abstract

Gastric cancer is the fourth leading cause of cancer deaths worldwide. Most patients are diagnosed in the advanced stage. Inadequate therapeutic strategies and the high recurrence rate lead to the poor 5-year survival rate. Therefore, effective chemopreventive drugs for gastric cancer are urgently needed. Repurposing clinical drugs is an effective strategy for discovering cancer chemopreventive drugs. In this study, we find that vortioxetine hydrobromide, an FDA-approved drug, is a dual JAK2/SRC inhibitor, and has inhibitory effects on cell proliferation of gastric cancer. Computational docking analysis, pull-down assay, cellular thermal shift assay (CETSA) and in vitro kinase assays are used to illustrate vortioxetine hydrobromide directly binds to JAK2 and SRC kinases and inhibits their kinase activities. The results of non-reducing SDS-PAGE and Western blotting indicate that vortioxetine hydrobromide suppresses STAT3 dimerization and nuclear translocation activity. Furthermore, vortioxetine hydrobromide inhibits the cell proliferation dependent on JAK2 and SRC and suppresses the growth of gastric cancer PDX model in vivo. These data demonstrate that vortioxetine hydrobromide, as a novel dual JAK2/SRC inhibitor, curbs the growth of gastric cancer in vitro and in vivo by JAK2/SRC-STAT3 signaling pathways. Our results highlight that vortioxetine hydrobromide has the potential application in the chemoprevention of gastric cancer.

## Introduction

Gastric cancer (GC) requires over one million new cancer cases in 2020 and an estimated 769,000 cancer-related deaths [1]. Most patients (>70%) developed to an advanced stage due to a lack of specific signs [2]. Chemoprevention is a promising strategy to improve GC patient 5-year survival rate. Accumulating evidence has illustrated that antipsychotics, anti-inflammatory drugs and antidepressants execute chemopreventive activities in GC [3, 4]. Non-steroidal anti-inflammatory drugs (NSAIDs), particularly aspirin, exerts chemopreventive effects in GC chemoprevention through COX-1 and COX-2 inhibition [3]. Fluoxetine induces apoptosis and autophagy in gastric cancer cells [5]. Therefore, screening effective chemopreventive drugs is of great significance for gastric cancer chemoprevention.

Tyrosine-protein kinase 2 (JAK2) and proto-oncogene tyrosine-protein kinase (SRC) are non-receptor protein tyrosine kinases. JAK2, as a signaling hub, integrates extracellular signals from oncogenic receptor tyrosine kinases and interleukin receptors to signal transducer and activator of transcription 3 (STAT3) [6]. SRC promotes cell proliferation, differentiation, survival, migration, invasion and angiogenesis by regulating Ras/Raf/ERK1/2, PI3K/Akt and STAT3 signaling pathways [7, 8]. JAK2 and SRC have emerged as important therapeutic targets for several cancers including head and neck squamous cell carcinoma, breast cancer, lung cancer and gastric cancer [7, 9–13]. Therefore, JAK2/SRC-based targeted therapies have broad therapeutic potential for GC treatment or chemoprevention.

Repurposing drugs is a time-saving way of developing chemopreventive drugs with higher efficacy and fewer side effects. Our group’s previous studies have proved that repurposed FDA-approved drugs can be used for the chemoprevention of esophageal squamous cell carcinoma (ESCC). Nuplazid suppresses ESCC growth by targeting PAK4. Tegaserod Maleate inhibits the ESCC cell proliferation through suppressing the peroxisome pathway. Cloperastine constrains ESCC growth via mitochondrial oxidative phosphorylation [14–16]. These results indicate that repurposing drugs for cancer chemoprevention is a promising strategy. In this study, we found that vortioxetine hydrobromide is a dual JAK2/SRC inhibitor and suppresses the growth of gastric cancer cells in vivo and in vitro.

## Results

### Vortioxetine hydrobromide inhibited GC cell proliferation in vitro

**Fig. 1:**
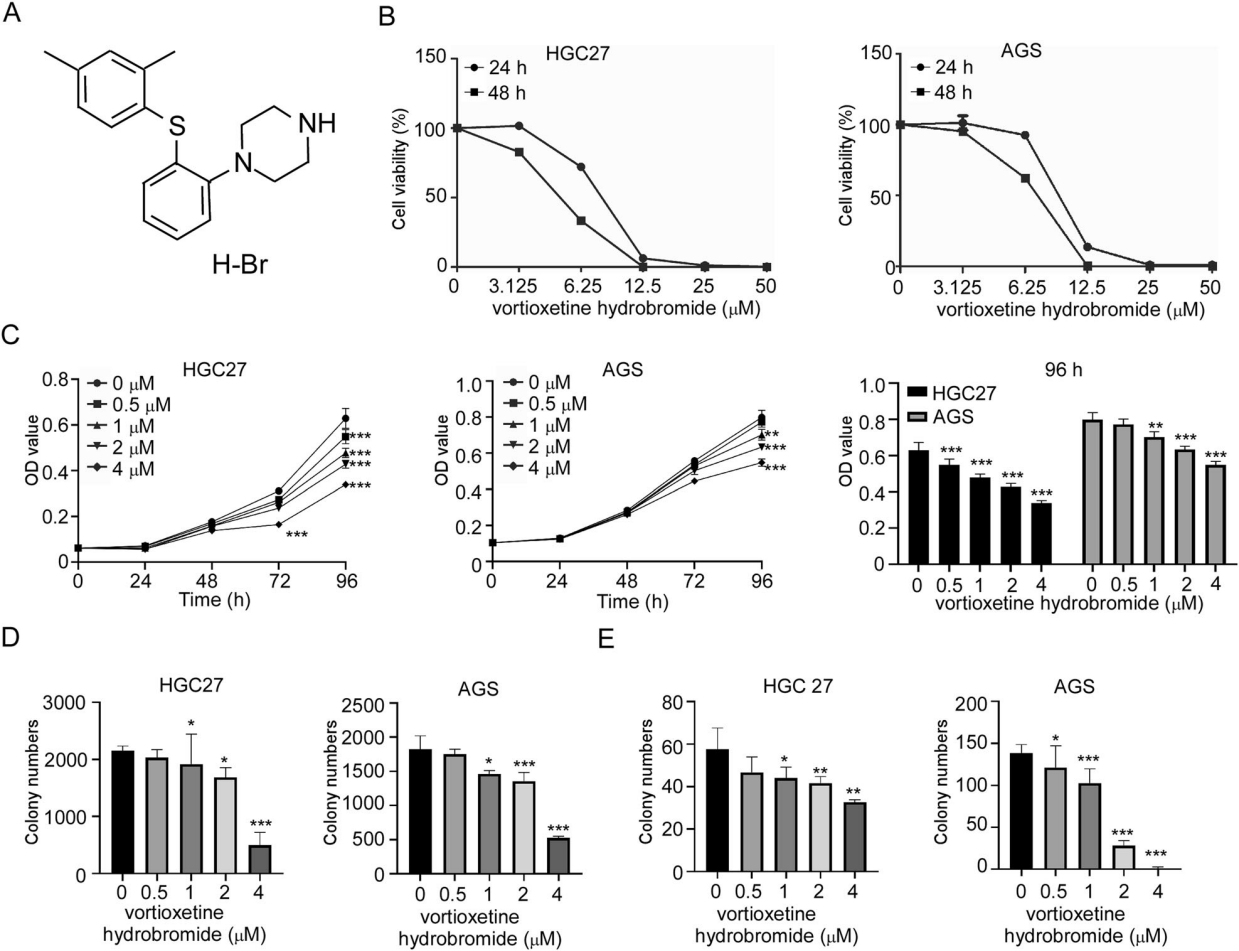
Vortioxetine hydrobromide inhabited GC cell proliferation in vitro. **A** The chemical structure of vortioxetine hydrobromide. **B** HGC 27 and AGS cells were seeded in 96-well plates and treated with vortioxetine hydrobromide (0, 3.125, 6.25, 12.5, 25 and 50 μM) for 24 and 48 h. The Y axis showed the corresponding relative cell viability. **C** The cells were treated with various concentrations of vortioxetine hydrobromide (0, 0.5, 1, 2, 4 μM) for 24, 48, 72 and 96 h. Cell viability was evaluated by MTT assay and normalized to that of the control. Mean ± S.D. (*n* = 3). **D** Effect of vortioxetine hydrobromide on anchorage-independent growth of GC cells. The cells (8 × 10^3^ cells/well) were treated with various concentrations of vortioxetine hydrobromide (0, 0.5, 1, 2, 4 μM) in an 1.25% basal medium eagle agar matrix containing 10% FBS and cultured for 9 days. Colony numbers were counted using the IN Cell Analyzer 6000 software. **E** The cells were plated into 6-well plates and treated with various concentrations of vortioxetine hydrobromide (0, 0.5, 1, 2, 4 μM) for 10 days, followed by crystal violet staining to monitor colony formation. Mean ± S.D. (*n* = 3) (*p < 0.05, **p < 0.01, ***p < 0.001).

The chemical structure of vortioxetine hydrobromide was shown in Fig. 1A. The half maximal inhibitory concentration (IC50) values of vortioxetine hydrobromide in HGC27 and AGS cells were 5.90 and 9.40 μM at 24 h, and 4.96 and 6.56 μM at 48 h (Fig. 1B). To determine the effects of vortioxetine hydrobromide on GC cell proliferation, HGC27 and AGS cells were treated with different concentrations of vortioxetine hydrobromide (0, 0.5, 1, 2 and 4 μM). The proliferation inhibitory rates of HGC27 and AGS were 66 and 45% after treatment with 4 μM vortioxetine hydrobromide for 96 h (Fig. 1C). Next, we investigated whether vortioxetine hydrobromide could inhibit anchorage-independent growth and clone formation of GC cells or not. The results of soft agar assay and clone formation assay illustrated that vortioxetine hydrobromide significantly inhibited anchorage-independent growth (Figs. 1D and S1A) and anchorage dependent growth (Figs. 1E and S1B) of GC cells in a dose-dependent manner. Together, these results indicated that vortioxetine hydrobromide inhibited GC cell proliferation in vitro.

### Vortioxetine hydrobromide directly bound with JAK2 and SRC

**Fig. 2:**
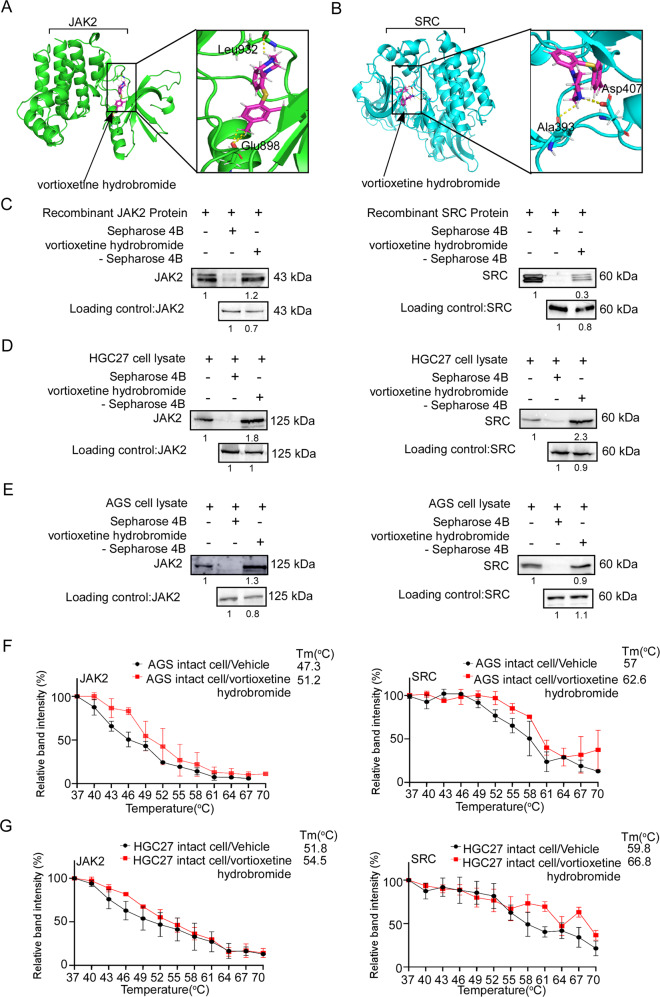
Vortioxetine hydrobromide bound with JAK2 and SRC. **A** The interaction between vortioxetine hydrobromide (pink) and JAK2 (green) was predicted using a computational docking model. Hydrogen bonds were represented as a yellow dash line. **B** The interaction between vortioxetine hydrobromide (pink) and SRC (blue) was predicted using a computational docking model. Hydrogen bonds were represented as a yellow dash line. **C**–**E** Vortioxetine hydrobromide directly bound to JAK2 and SRC. The recombinant proteins (**C**) or GC cell lysates (**D**, **E**) were incubated with vortioxetine hydrobromide-conjugated Sepharose 4B beads or with Sepharose 4B beads alone. The results were analyzed by Western blotting. Data from three independent experiments were shown. The binding capacity of vortioxetine hydrobromide to JAK2 and SRC in AGS (**F**) and HGC27 (**G**) intact cells. The cells were treated with vortioxetine hydrobromide or DMSO for 24 h and incubated in different temperatures. The protein bindings were visualized by Western blotting. Mean ± S.D. (*n* = 3).

To further investigate the molecular mechanism of vortioxetine hydrobromide on gastric cancer, we used Maestro 11.5 software to conduct computational docking models. We found JAK2 and SRC were potential targets of vortioxetine hydrobromide (Fig. 2A, B). Then pull-down assays were performed using recombinant JAK2 or SRC proteins, HGC27 and AGS cell lysates. The results demonstrated that the vortioxetine hydrobromide directly bound to JAK2 or SRC proteins (Fig. 2C–E). What’s more, we made predictions through the SwissTargetPrediction website, and the results showed that EGFR, mTOR and AKT kinases were likely to bind to vortioxetine hydrobromide. However, the results of pull-down assays indicated that EGFR, mTOR and AKT could not bind with vortioxetine hydrobromide (Fig. S2A). Even, we evaluated the IC50 of vortioxetine hydrobromide with JAK2 kinase or Src kinase by Kinase-Lumi™ luminescent kinase assay. The IC50 of vortioxetine hydrobromide with SRC or JAK2 was 6.497 and 8.529 μM (Fig. S2B), respectively. To further verify the interactions between vortioxetine hydrobromide and JAK2 or SRC kinase in intact cells, we performed cellular thermal shift assay. The melting temperature (Tm) values of JAK2 and SRC in AGS cell were 51.2 and 62.6 °C after vortioxetine hydrobromide treatment, which were higher than 47.3 and 57 °C in vehicle groups (Figs. 2F and S2C). Similarly, the Tm values of JAK2 and SRC in vortioxetine hydrobromide-treated group were 54.5 and 66.8 °C, while the vehicle group were 51.8 and 59.8 °C (Figs. 2G and S2D). These results confirmed that vortioxetine hydrobromide bound with JAK2 and SRC in intact cells. Taken together, vortioxetine hydrobromide directly bound with JAK2 and SRC kinases.

### Vortioxetine hydrobromide inhibited STAT3 signaling pathway by targeting JAK2 and SRC kinases

**Fig. 3:**
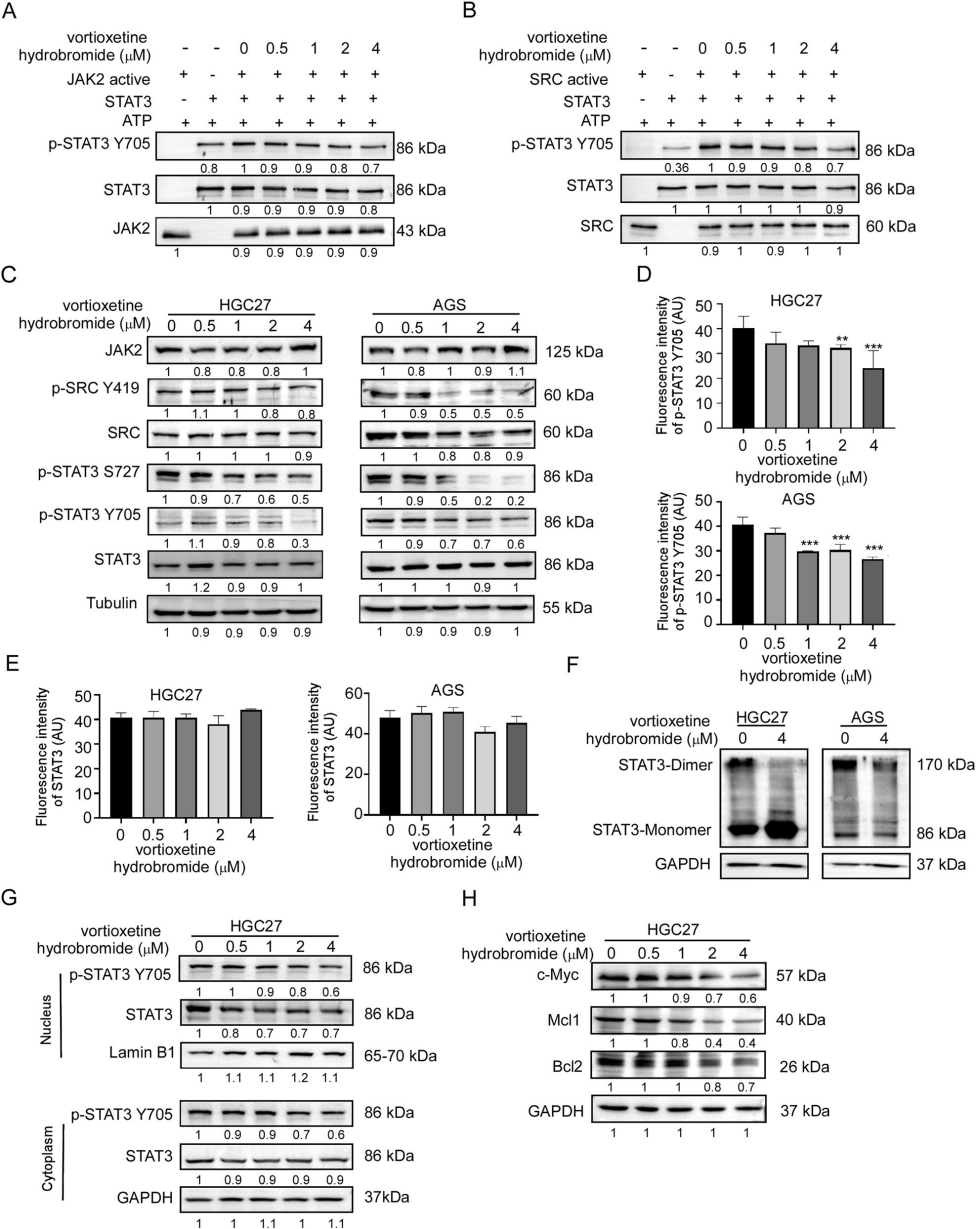
Vortioxetine hydrobromide suppressed STAT3 signaling pathway by targeting JAK2 and SRC kinases. **A** In vitro kinase assay of active JAK2 and inactive STAT3. The active JAK2, inactive STAT3, and ATP mixture were treated with vortioxetine hydrobromide or DMSO at 30 °C for 30 min. p-STAT3 Y705 and STAT3 were visualized by Western blotting. **B** Vortioxetine hydrobromide suppressed SRC kinase activity in a dose-dependent manner. **C** The protein levels of JAK2/SRC-STAT3 signaling pathway in GC cells after vortioxetine hydrobromide (0, 0.5, 1, 2, 4 μM) treatment. The quantitative analyses of fluorescence intensity of p-STAT3 (**D**) and STAT3 (**E**) in GC cells. **F** The changes of STAT3 dimer formation after vortioxetine hydrobromide treatment in GC cells. **G** The nucleus localization variation of STAT3 after treatment of various concentrations of vortioxetine hydrobromide in HGC27 cells. **H** The protein levels of Bcl2, Mcl1 and c-Myc by Western blotting after treatment of various concentrations of vortioxetine hydrobromide in HGC27 cells. Mean ± S.D. (*n* = 3) (*p < 0.05, **p < 0.01, ***p < 0.001).

Previous studies have illustrated that STAT3 proteins can be activated by JAK2 and SRC kinases [6, 8]. In vitro kinase assay was performed to detect the inhibitory effects of vortioxetine hydrobromide on JAK2 and SRC kinase activities. Results demonstrated that vortioxetine hydrobromide inhibited the activities of JAK2 and SRC kinases (Fig. 3A, B). Next, we determined whether the JAK2/SRC-STAT3 signaling pathways in GC cells could be regulated by vortioxetine hydrobromide. Western blotting results confirmed that vortioxetine hydrobromide suppressed STAT3 phosphorylation in a dose-dependent manner (Fig. 3C). Immunofluorescence assays also demonstrated that phosphorylation of STAT3 were inhibited by 40% and 34% in HGC27 and AGS cells at 4 μM vortioxetine hydrobromide, respectively (Fig. 3D), whereas total STAT3 remained unchanged (Figs. 3E and S3A). It has been reported that STAT3 protein is phosphorylated at the conserved tyrosine residue (Tyr705), resulting in STAT3 dimerization and nuclear translocation [17, 18]. Non-reducing SDS-PAGE was performed to evaluate the change in STAT3 dimerization. Results demonstrated that STAT3 dimerization decreased in GC cells after vortioxetine hydrobromide treatment (Fig. 3F). Furthermore, after the vortioxetine hydrobromide treatment, the protein levels of p-STAT3 and STAT3 in HGC27 cell nucleus (Fig. 3G) and AGS cell nucleus (Fig. S3B) decreased. Subsequently, the protein levels of STAT3-mediated downstream substrates including Bcl-2, Mcl-1 and c-Myc were impaired by vortioxetine hydrobromide in HGC27 (Fig. 3H) and AGS cells (Fig. S3C). Vortioxetine hydrobromide reduced Bcl-2, Mcl-1 and c-Myc protein levels, inhibiting GC cell proliferation and survival. Thus, the above results suggested that vortioxetine hydrobromide inhibited GC by blocking the JAK2/SRC-STAT3 pathways.

### JAK2 or SRC knockout suppressed GC cell proliferation and reduced the sensitivity of GC to vortioxetine hydrobromide

In order to assess the prognostic and potential therapeutic value of JAK2 and SRC in GC patients, the mRNA expressions of JAK2 and SRC were analyzed by UALCAN. The bioinformatic analysis confirmed that JAK2 and SRC mRNA levels were up-regulated in a variety of cancers (Fig. S4A, B). It was observed that JAK2 and SRC mRNA levels were higher in GC tissues compared to normal tissues (Fig. S4C, E), and higher in G1, G2 and G3 grade compared to normal tissues (Fig. S4D, F). These data suggested that JAK2 and SRC were up-regulated in GC and their mRNA levels were positively correlated with poor clinical grades.

**Fig. 4:**
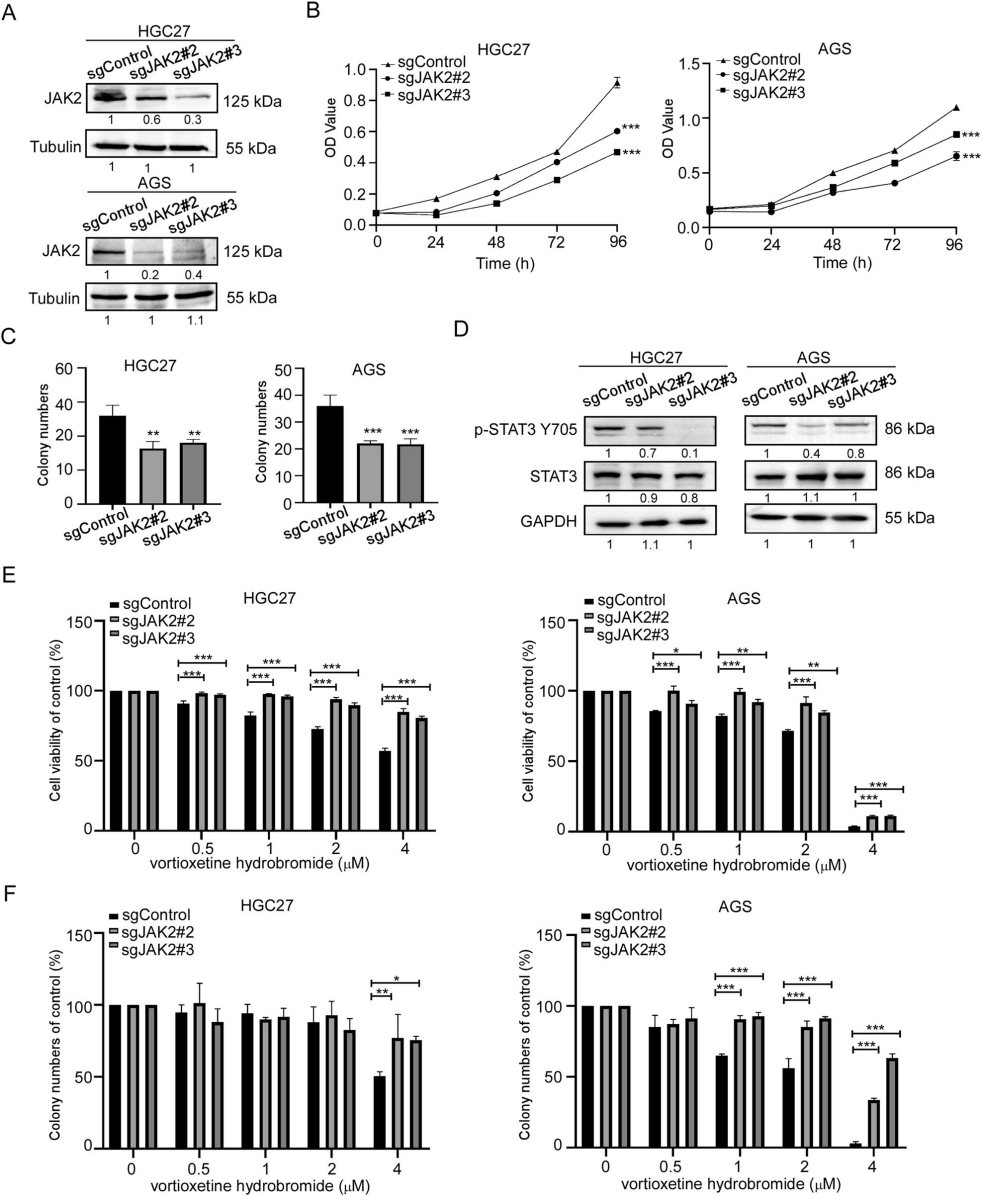
JAK2 knockout reduced GC cells sensitivity to vortioxetine hydrobromide. **A** CRISPR/Cas9 system was used to knockout JAK2 in HGC27 and AGS cells. Knockout efficiency in GC cells was assessed by Western blotting. **B** Cell viability after JAK2 knockout was assessed by MTT (96 h after seeding cells) assay. **C** Colony numbers of JAK2 knockout cells were measured. **D** The protein levels of p-STAT3 Y705 and STAT3 in JAK2 knockout cells by Western blotting. **E** The inhibitory effect of vortioxetine hydrobromide on JAK2 knockout cells was detected by proliferation assay after 96 h. Cell viability was evaluated by MTT assay and normalized to that of the sgcontrol. **F** JAK2 knockout cells were plated into 6-well plates and treated with various concentration of vortioxetine hydrobromide (0, 0.5, 1, 2, 4 μM) for 10 days, followed by crystal violet staining to monitor colony formation. Mean ± S.D. (*n* = 3) (*p < 0.05, **p < 0.01, ***p < 0.001).

**Fig. 5:**
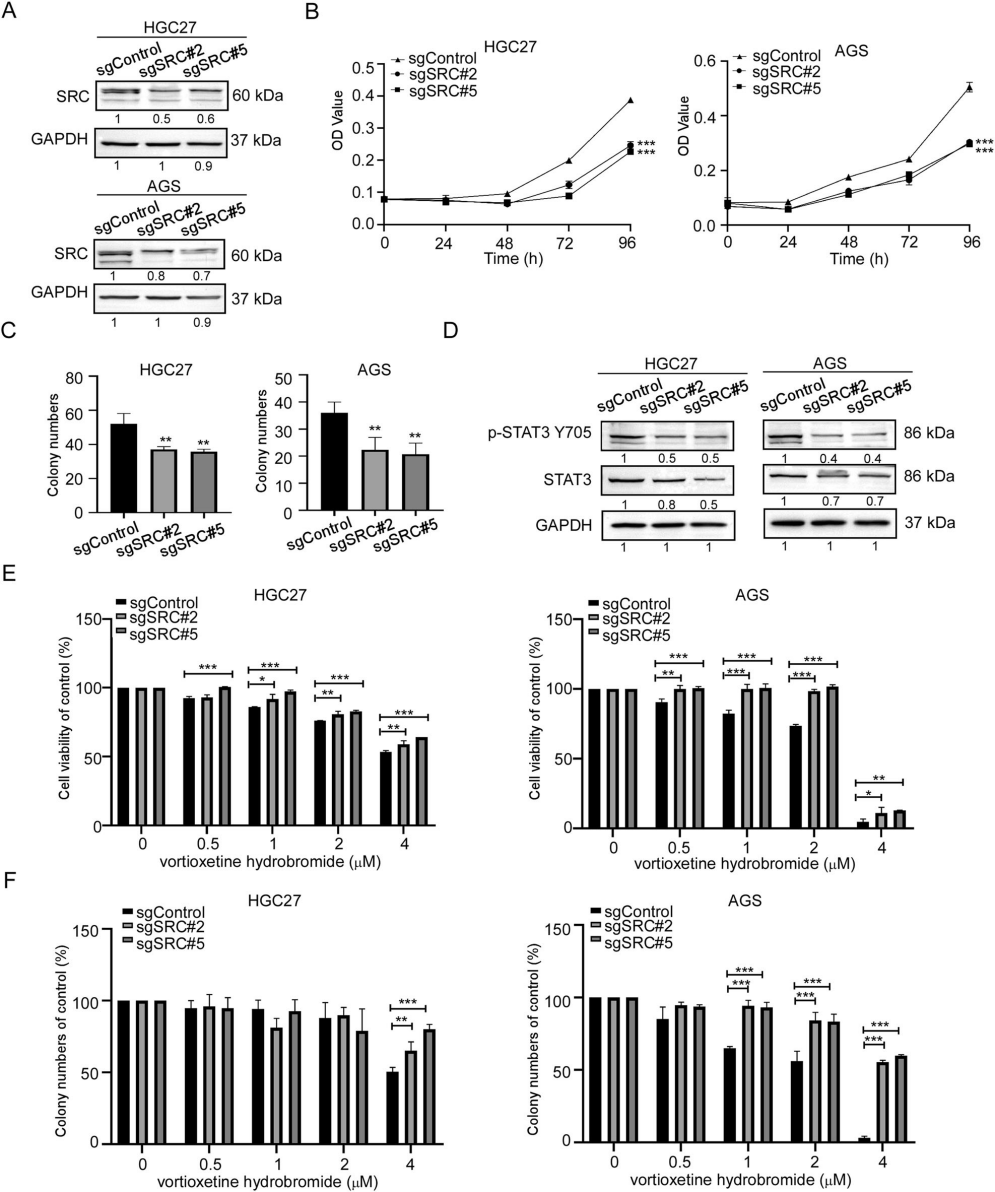
SRC knockout reduced GC cells sensitivity to vortioxetine hydrobromide. **A** CRISPR/Cas9 system was used to knockout SRC in HGC27 and AGS cells. Knockout efficiency in GC cells were assessed by Western blotting. **B** Cell viability after SRC knockout was assessed by MTT (96 h after seeding cells) assay. **C** Colony numbers of SRC knockout cells were measured. **D** The protein levels of p-STAT3 Y705 and STAT3 in SRC knockout cells by Western blotting. **E** The inhibitory effect of vortioxetine hydrobromide on SRC knockout cells was detected by proliferation assay after 96 h. Cell viability was evaluated by MTT assay and normalized to that of the control. **F** SRC knockout cells were plated into 6-well plates and treated with various concentration of vortioxetine hydrobromide (0, 0.5, 1, 2, 4 μM) for 10 days, followed by crystal violet staining to monitor colony formation. Mean ± S.D. (*n* = 3) (*p < 0.05, **p < 0.01, ***p < 0.001).

To investigate the role of JAK2 or SRC in GC growth, we established JAK2 or SRC knockout cell lines by CRISPR/Cas9 system. We verified the knockout efficiency of JAK2 or SRC by Western blotting, respectively. Results showed that the protein levels of JAK2 or SRC in sgJAK2#2, sgJAK2#3, sgSRC#2 and sgSRC#5 cells decreased (Figs. 4A and 5A). After knockout JAK2 or SRC, the growths of GC cells were suppressed (Figs. 4B and 5B). Similarly, the colony-formation ability of sgJAK2 and sgSRC cells were significantly attenuated (Figs. 4C, 5C and S5A). Hence, the phosphorylation at Y705 of STAT3 in sgJAK2 and sgSRC cells decreased (Figs. 4D and 5D). To evaluate whether the effects of vortioxetine hydrobromide were dependent upon JAK2 and SRC expression, we performed cell proliferation assay and colony formation assay in sgJAK2 and sgSRC cells treated with vortioxetine hydrobromide. The results confirmed the inhibitory effect of vortioxetine hydrobromide on the proliferation and colony formation of sgJAK2 (Fig. 4E, F) and sgSRC cells (Figs. 5E, F and S5B) decreased compared to the sgControl cells. We also constructed JAK2 or SRC knockout monoclonal cell lines. We found JAK2 or SRC knockout monoclonal cells had lower levels of JAK2 or SRC (Fig. S6A) in GC cells. After knocking out JAK2 or SRC, the proliferation and colony-formation abilities decreased (Fig. S6B, C). Next, JAK2 or SRC knockout monoclonal cells were treated with 4 μM vortioxetine hydrobromide, the sensibility of monoclonal cells to vortioxetine hydrobromide was less than wild type cells (Fig. S6D, E).

**Fig. 6:**
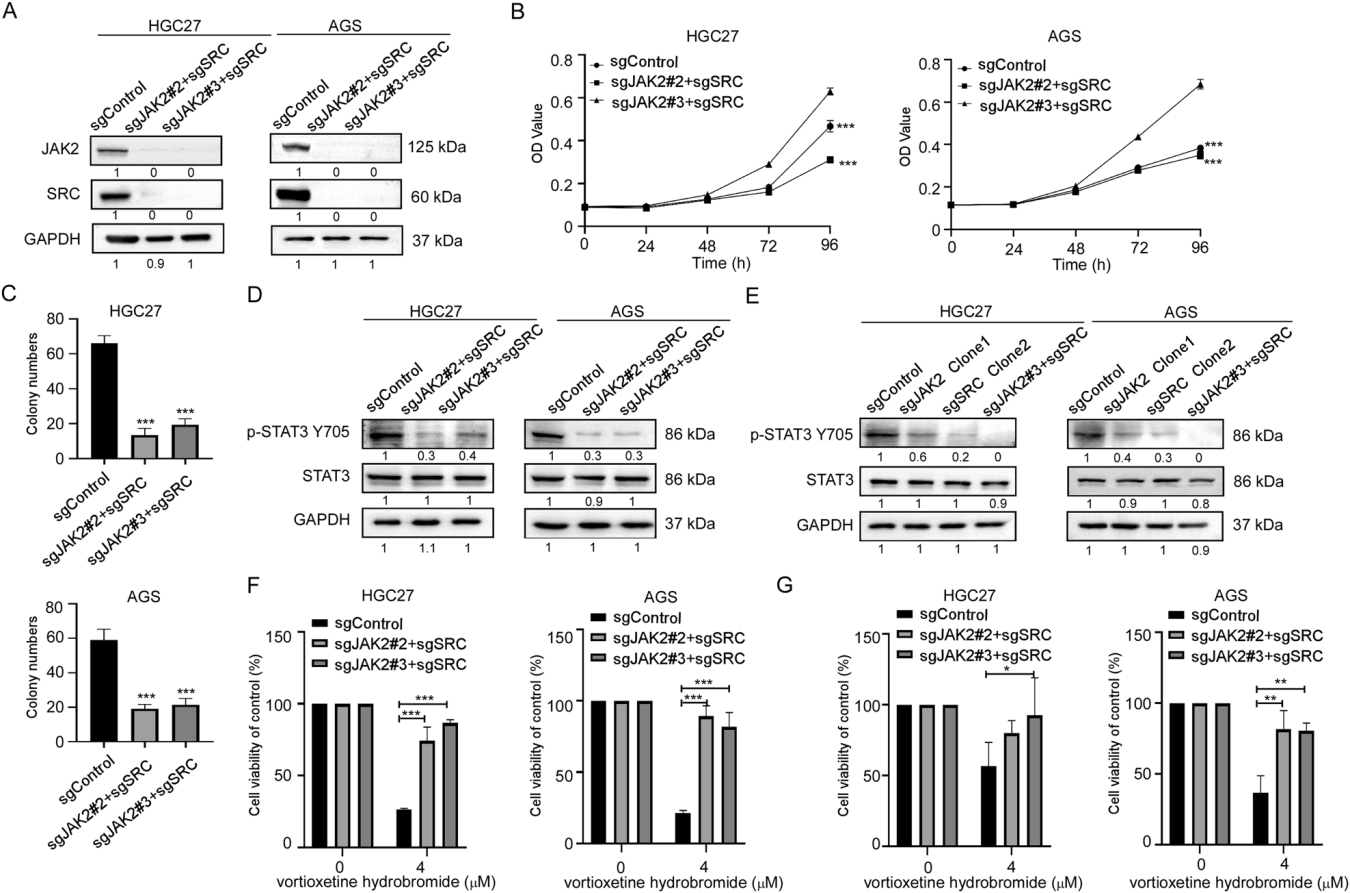
Knockout JAK2 and SRC reduced GC cells' sensitivity to vortioxetine hydrobromide. **A** CRISPR/Cas9 system was used to knockout both JAK2 and SRC in HGC27 and AGS cells. Knockout efficiency in GC cells were assessed by Western blotting. **B** Cell viability after both JAK2 and SRC knockout was assessed by MTT assay. **C** Colony numbers of JAK2 and SRC knockout cells were measured by colony formation assay. **D** The protein levels of p-STAT3 Y705 and STAT3 in JAK2 and SRC double knockout cells by Western blotting. **E** The protein levels of p-STAT3 Y705 and STAT3 in sgControl, sgJAK2, sgSRC and sgJAK2 + sgSRC cells by Western blotting. **F** The inhibitory effect of vortioxetine hydrobromide on JAK2 and SRC knockout cells was detected by proliferation assay after 96 h. Cell viability was evaluated by MTT assay and normalized to that of the sgcontrol. **G** JAK2 and SRC knockout cells were plated into 6-well plates and treated with 4 μM vortioxetine hydrobromide for 10 days, followed by crystal violet staining to monitor colony formation. Mean ± S.D. (*n* = 3) (*p < 0.05, **p < 0.01, ***p < 0.001).

We further established the dual JAK2 and SRC knockout GC cells. The results showed that the protein levels of both JAK2 and SRC in sgJAK2#2+sgSRC and sgJAK2#3+sgSRC cells decreased (Fig. 6A). Then, we investigated whether dual knockout of both JAK2 and SRC would inhibit growth of GC cells or not. The results of cell proliferation assays and colony formation assays confirmed that the growth (Fig. 6B) and colony formation abilities (Figs. 6C and S7A) of the both JAK2 and SRC knockout cells were significantly reduced compared with the sgControl cells. And the levels of p-STAT3 Y705 in the dual knockout cells were greatly reduced (Fig. 6D). What’s more, we compared the p-STAT3 Y705 levels of sgControl, sgJAK2, sgSRC and sgJAK2+sgSRC cells in the same graph by Western blotting. Results showed that the protein level of p-STAT3 Y705 decreased gradiently from the sgControl, sgJAK2 and sgSRC cells to sgJAK2 + sgSRC cells (Fig. 6E). Followly, we treated JAK2 and SRC dual knockout cells with vortioxetine hydrobromide, the inhibitory effect of vortioxetine hydrobromide on the proliferation of JAK2 and SRC dual knockout cells decreased compared with the sgControl cells by MTT assay (Fig. 6F) and clone formation assay (Figs. 6G and S7B). Together, these results illustrated that vortioxetine hydrobromide suppressed GC growth through JAK2 and SRC kinases.

### Vortioxetine hydrobromide inhibited the growth of GC patient-derived xenograft (PDX)

**Fig. 7:**
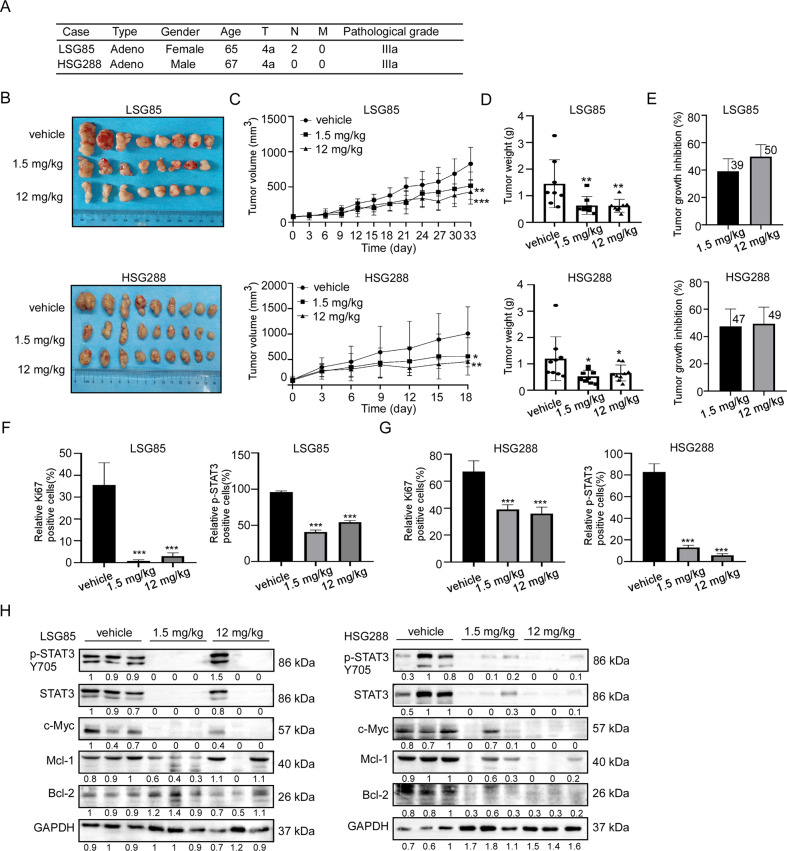
Vortioxetine hydrobromide inhibited the growth of patient-derived xenograft (PDX) tumor in vivo. **A** Patient information of different PDX cases: type, gender, age, clinical phases and pathological grade. **B** HSG 288 and LSG85 SCID mice received 1.5 mg/kg and 12 mg/kg vortioxetine hydrobromide once a day. Photographs of tumors for LSG85 (*n* = 8) and HSG 288 (*n* = 9). **C** Tumor volumes were measured every 3 days. Tumor growth curve after vortioxetine hydrobromide treated for LSG85 (*n* = 8) and HSG 288 (*n* = 9). **D** Tumor weights were measured every 2 days. **E** The tumor growth inhibition of vortioxetine hydrobromide. Statistical analysis of Ki67 and p-STAT3 Y705 protein levels in LSG85 (**F**) and HSG288 (**G**). Vortioxetine hydrobromide-treated groups were normalized to the vehicle group. **H** The protein levels of p-STAT3 Y705, STAT3, Bcl2, Mcl1 and c-Myc of tumor tissues were detected by Western blotting. Mean ± S.D. (*n* = 3) (*p < 0.05, **p < 0.01, ***p < 0.001).

In order to evaluate the anti-tumor activity of vortioxetine hydrobromide in vivo, we established LSG85 and HSG288 PDX models in mice. The information of patients was described in Fig. 7A. Vortioxetine hydrobromide inhibited the growth of GC PDX models (LSG85 and HSG288 cases) following 33-day and 18-day of treatment with 1.5 or 12 mg/kg by gavage every day (Fig. 7B). The tumor volumes and weights of vortioxetine hydrobromide groups in both cases decreased compared with the vehicle groups (Fig. 7C, D). The tumor sizes of individual mice in LSG85 and HSG288 cases were shown in Fig. S8A. We calculated tumor weights and found the average tumor growth inhibitory rate of 12 mg/kg groups in the two cases were 50% and 49% (Fig. 7E), respectively. Next, the effect of vortioxetine hydrobromide on the protein levels of Ki67 and p-STAT3 Y705 in tumor tissues were determined by immunohistochemistry (IHC). The results illustrated vortioxetine hydrobromide significantly inhibited the protein levels of Ki67 and p-STAT3 Y705 compared with the vehicle groups in LSG85 case (Figs. 7F and S8B) and HSG288 case (Figs. 7G and S8B). These data indicated that vortioxetine hydrobromide inhibited tumor proliferation and progression. Western blotting results confirmed that vortioxetine hydrobromide reduced the levels of p-STAT3 Y705, c-Myc, Bcl-2 and Mcl-1 in PDX tumor xenografts (Fig. 7H). Meanwhile, there were no obvious toxic effects on the organs (heart, liver, spleen, lung, kidney and brain) (Fig. S8C), and there were no significant changes in body weights (Fig. S8D). All together, these data suggested that vortioxetine hydrobromide inhibited GC PDX growth in vivo.

## Discussion

Currently, the main treatment methods for gastric cancer patients include surgery, chemotherapy, radiotherapy and immunotherapy [19–21]. However, the high recurrence and metastasis after surgery lead to the poor 5-year survival rate, and the toxic effects of chemoradiotherapy limit the use of these regimens for GC treatment [22, 23]. Therefore, it is necessary to find effective chemopreventive drugs for gastric cancer recurrence prevention. Repurposing FDA-approved drugs is an effective way to prevent cancer, because drugs have established safety, pharmacokinetics, tolerability and toxicity profile [14]. In this study, we found that vortioxetine hydrobromide, an FDA-approved drug, had anticancer effects on GC cell proliferation in vitro and GC PDX growth in vivo. This evidence further suggests that vortioxetine hydrobromide is a promising drug for GC chemoprevention.

JAK2 and SRC overexpression or overactivation are closely related to the development, maintenance and progression of cancers, suggesting that JAK2 and SRC are effective targets for cancer therapy [7, 11, 24]. JAK2 mediates multiple aspects of cytokine signaling, leading to supporting survival and promoting proliferation in breast cancer and B-cell leukemias and lymphomas [25, 26]. Excessive activation of SRC kinase increases cell adhesion, invasion, epithelial to mesenchymal transition (EMT), and migration in colon cancer, pancreatic cancer and breast cancer [7, 27, 28]. Our data showed that knocking out JAK2 and SRC could inhibit the proliferation of GC cells, and reduced the sensitivity of GC cells to vortioxetine hydrobromide treatment. Furthermore, vortioxetine hydrobromide directly bound with JAK2 and SRC kinase. Therefore, vortioxetine hydrobromide is a dual JAK2/SRC inhibitor. It has been reported that STAT3 could be activated by JAK2 and SRC kinases [6, 8]. STAT3 is regarded as a therapeutic target in many cancers and regulates cyclinD1, c-Myc, Bcl-2, survivin and ICAM-1, related to cell proliferation, survival, migration, invasion, stemness, immunosuppression and angiogenesis [6, 18, 29–31]. In this study, vortioxetine hydrobromide inhibited the activities of JAK2 and SRC kinases, through reducing STAT3 dimerization and nuclear translocation. Recently, it has been reported that vortioxetine hydrobromide can inhibit the cell proliferation of AGS through PI3K/AKT signaling pathway, but no specific target of this drug has been confirmed [32]. This evidence illustrated that vortioxetine hydrobromide suppressed JAK2/SRC-STAT3 signaling pathways.

In the past 5 years, the use of dual or multi-target inhibitors as new therapeutic ways for anti-tumor studies has been steadily increasing [33]. Dual kinase inhibitors, Dasatinib and Lapatinib, have shown increased efficacy for inhibiting SRC/ABL and EGFR/HER2, respectively [34, 35]. Indirubin derivative (IRD) E738, NS-018 and SKLB-850, dual JAK2/SRC inhibitors, have demonstrated inhibitory effects in pancreatic cancer, multiple myeloma and B-cell lymphoma [36–38]. These inhibitors are newly developed small molecules and are still in preclinical research. Of note, IRD E738, NS-018 and SKLB-850 are not approved by FDA yet. While, vortioxetine hydrobromide is approved by FDA, and acts as an anti-depressant by inhibiting serotonin transporter (SERT) and modulating 5-HT receptor activity [39]. Although the sensitivity of vortioxetine hydrobromide is weak comparing to Dasatinib and Tofacitinib [40, 41], vortioxetine hydrobromide, as a dual-target inhibitor, can greatly reduce the financial burden of patients. And our data showed that there were no obvious toxic effects on the mice organs and no significant changes in body weights (Fig. S8). In addition, the incidence of depression in cancer patients is high [42]. Vortioxetine hydrobromide may have a better clinical therapeutic effect for gastric cancer patients with depression or anxiety. Therefore, our research implies vortioxetine hydrobromide is a promising chemopreventive agent or drug for adjuvant therapy of GC.

In conclusion, we identified vortioxetine hydrobromide, a novel dual JAK2/SRC inhibitor, inhibited gastric cancer cell proliferation, through reducing STAT3 dimerization and nuclear translocation in vivo and in vitro.

## Methods

### Cell culture

Human GC cell lines HGC27 and AGS cells were purchased from the Chinese Academy of Sciences Cell Bank (Shanghai, China). All the cell lines were cytogenetically by STR analysis. HGC27 cells were cultured in RPMI-1640 medium and AGS cells were cultured in F12K containing 10% fetal bovine serum (FBS) at 37 °C in 5% CO_2_.

### Chemicals and reagents

Vortioxetine hydrobromide (CAS number 960203-27-4) was purchased from Beijing Bailing Wei Technology Co. Ltd. The primary antibodies p-STAT3 Y705 (9145S), p-STAT3 S727 (9134S), STAT3 (9139S), JAK2 (3230S), p-JAK2 1007/1008 (3771S), SRC (2108S) were purchased from Cell Signaling Technology (Danvers, MA, USA). p-SRC Y419 (ab185617), recombinant JAK2 protein (ab42619), Ki67 (ab16667) were purchased from Abcam (Cambridge, MA, USA). STAT3 (sc-8019), p-STAT3 Y705 (sc-8059), Mcl-1 (sc-12756) and Bcl-2 (sc-7382) were from Santa Cruz (Dallas, TX, USA); GAPDH was from Good Here Biological Technology Co. Ltd. (Hangzhou, Zhejiang, China). The secondary antibodies anti-rabbit IgG (ZB-2301) and anti-mouse IgG (ZB-2305) were from Zhongshan Jinqiao (Beijing, China). The Kinase-Lumi™ chemiluminescent kinase activity detection kit (S0158S) were from Beyotime (Shanghai, China).

### Cytotoxicity assay

HGC27 (6000 cells/well) and AGS (8000 cells/well) were plated on 96-well plates and treated with different doses of vortioxetine hydrobromide (0, 3.125, 6.25, 12.5, 25, 50 μM). After 24, 48 h, the cells were fixed with 4% paraformaldehyde for 30 min at room temperature. Then the cells were stained with DAPI (1:10,000) in the dark and counted using IN Cell Analyzer 6000 software.

### MTT assay

GC cells (3000 cells/well) were plated on 96-well plates and treated with different doses of vortioxetine hydrobromide (0, 0.5, 1, 2, 4 μM). Plates were taken out at 0, 24, 48, 72 or 96 h. 10 µL of MTT (5 mg/ml) was added to each well and cells were incubated at 37 °C for 2–3 h. The medium was then aspirated from the plates and replaced with 100 µL of DMSO. The absorbance at 490 nm was measured using a microplate reader.

### Anchorage-independent cell growth

Eagle’s basal medium (BME) with 0.4% agar was added to each well of a 6-well plate and solidified at room temperature for 1.5 h. HGC 27 and AGS cells (8000 cells/well) were suspended in BME containing 0.2% agar with different doses of vortioxetine hydrobromide (0, 0.5, 1, 2 and 4 μM). After incubation for 2 h at room temperature, cells were maintained in a 37 °C, 5% CO_2_ incubator for 9 days, and then the clones were counted and scanned using IN Cell Analyzer 6000 software.

### Colony formation assay

GC cells (300 cells/well) were seeded into 6-well plates and treated with different doses of vortioxetine hydrobromide (0, 0.5, 1, 2 and 4 μM). The medium was replenished with fresh medium containing vortioxetine hydrobromide every 3 days. After incubation for 10 days, the medium was then aspirated from the plates. The clones were fixed and then stained with 0.3% crystal violet. Then the clones were imaged and counted.

### Computational docking analysis

In silico docking was performed using the Maestro 11.5 software program. The crystal structures of JAK2 (PBD: 3E63) and SRC (PBD: EKSW) were obtained from the Protein Data Bank (PDB) (https://www.rcsb.org/). The vortioxetine hydrobromide chemical structure (PubChem CID: 9966051) was obtained from PubChem (https://pubchem.ncbi.nlm.nih.gov/). All water molecules and small molecule ligands were deleted and hydrogen atoms were added. The PyMOL (PyMOL Molecular Graphics System, Version 2.3.4) program was utilized to prepare the vortioxetine hydrobromide for the docking study.

### Western blotting

Cells (4.5 × 10^6^) were plated on a 15 cm dish and treated with various concentrations of vortioxetine hydrobromide (0, 0.5, 1, 2 and 4 μM) for 24 h. Then the cells were collected and lysed with RIPA buffer. The protein concentration was detected using the BCA kit (BCA Protein Assay Kit, Beyotime Biotechnology, Shanghai, China). Protein samples were heated with protein loading buffer at 100 °C for 5 min and then were separated by SDS-PAGE and transferred to a PVDF membrane. The membrane was blocked with 5% nonfat milk for 1.5 h, incubated with primary antibodies overnight at 4 °C. Next, the membranes were incubated with corresponding secondary antibodies for 1.5 h and were detected using a chemiluminescence reagent (ECL, Meilunbio).

### Pull-down assay

Cell lysates (500 µg) or recombination proteins were incubated with vortioxetine hydrobromide-Sepharose 4B beads or DMSO-Sepharose 4B beads in reaction buffer at 4 °C overnight. The beads were washed three times with buffer at 4 °C. The proteins were eluted from the beads through boiling in loading buffer at 95 °C for 5 min. Protein binding was assessed using Western blotting.

### Cellular thermal shift assay (CETSA)

Cells (4.5 × 10^6^) were seeded into a 15 cm dish and treated with vortioxetine hydrobromide (0, 4 μM) for 24 h. Then they were harvested and resuspended in PBS. The cells were divided into 12 tubes equally. The control group and vortioxetine hydrobromide treatment group were heated at 37, 40, 43, 46, 49, 52, 55, 58, 61, 64, 67 or 70 °C for 3 min. Then the samples were quickly frozen twice in liquid nitrogen and centrifuged at 12,000 rpm for 20 min at 4 °C. The proteins were transferred to new tubes and then analyzed by Western blotting [43].

### In vitro kinase assay and Kinase-Lumi™ luminescent kinase assay

Active JAK2 (50 ng) or SRC (100 ng) recombination proteins were incubated with inactive STAT3 proteins (250 ng) for in vitro kinase assays separately. The reactions were run in kinase buffer and contained 100 μM ATP. Different concentrations of vortioxetine hydrobromide were added and the mixtures were incubated at 30 °C for 30 min. The reactions were terminated by adding 5 µL protein loading buffer and boiled for 5 min at 95 °C. The proteins were analyzed using Western blotting. For Kinase-Lumi™ luminescent kinase assay, more details were described in the manufacturer’s protocol (Beyotime Biotechnology Company, Shanghai, China) [44]. Different concentrations of vortioxetine hydrobromide (0, 1, 5, 10 and 50 μM) were added into the kinase reactions. The reaction was subsequently initiated by the addition of 50 µL kinase detection reagent. After incubation for 10 min at room temperature, then the luminescence value was measured by a multi-function microplate reader (Centro XS3 LB 960, San Jose, USA). The consumption of ATP was detected to evaluate the inhibitory of vortioxetine hydrobromide on the SRC and JAK2 kinase.

### Cell immunofluorescence assay

The slides were put in a 24-well plate, and then 2 × 10^4^ GC cells were seeded in each well. After 16–18 h, cells were treated with various concentrations of vortioxetine hydrobromide for 24 h. The medium was then aspirated from the plate and the cells were fixed with 4% paraformaldehyde in PBS for 30 min. The cells were washed three times with PBS and incubated primary antibody (p-STAT3 Y705, STAT3 1:50) overnight at 4 °C. Next, slides were incubated with the fluorescent secondary antibody (1:50) for 1.5–2 h in the dark. After several washes, slides were stained with DAPI (1:10,000) for 5 min at 37 °C. The images were captured and analyzed by IN Cell Analyzer 6000 software.

### Nuclear and cytoplasmic protein extraction

In total, 2 × 10^6^ cells were seeded into a 10 cm dish and treated with different concentrations of vortioxetine hydrobromide (0, 0.5, 1, 2 and 4 μM) for 24 h. After harvesting the cells, the nuclear and cytoplasmic protein extraction kit (Cat#R0050, Solarbio) was used to extract nuclear proteins and cytoplasm proteins. The proteins were assessed by Western blotting.

### JAK2 and SRC knockout cell lines

To knockout the JAK2 or SRC gene in GC cells using the CRISPR/Cas9 system, the lentiCRISPR v2 vector was used. Following the manufacturer’s suggested protocols, sgJAK2 or sgSRC plasmids were transfected into HEK293T cells using Jet Primer (Thermo Fisher Scientific, Waltham, MA, USA). Two days after transfection, viral particles were harvested and filtered using a 0.22-μm filter. The GC cells were infected with 8 μg/ml polybrene and selected with 2 μg/ml puromycin for 72 h. Knockout efficiency was verified by Western blotting. The oligonucleotide sequences of JAK2 and SRC single guide (sg) RNA were designed in an online CRISPR tool (https://chopchop.cbu.uib.no/) and were listed as Table 1.

**Table 1:** The oligonucleotide sequences of JAK2 and SRC single guide (sg) RNA.

Gene name	Primer sequences 5′‐3′
JAK2#2	F: CACCGAATGAAGAGTACAACCTCAG
	R: AAACCTGAGGTTGTACTCTTCATTC
JAK2#3	F: CACCGCTTCTAGTCTTCAGAACGAA
	R: AAACTTCGTTCTGAAGACTAGAAGC
SRC#2	F: CACCGCCGAGCCCAAGCTGTTCGG
	R: AAACCCGAACAGCTTGGGCTCGGC
SRC#5	F: CACCGACCTGGAACGGTACCACCA
	R: AAACTGGTGGTACCGTTCCAGGTC

*F* forward, *R* reverse.

### Monoclonal cell lines

SgJAK2 or sgSRC cells were diluted with RPMI-1640 to a concentration of 50–60 cells/ml steps by steps, then seeded on 96-well plate of 100 µL/well. After 7–10 days, the positive monoclones were selected. When the clones were formed, the cells were digested with trypsin. 10 µL cell suspension was added by 40 µL K-buffer (10 mM Tris-HCl, pH 8.0, 50 mM KCl, 1.5 mM MgCl_2_, 0.5% Tween-20, 0.1 mg/ml protease K), then incubated at 60 °C for 1 h. RT-PCR was performed with these samples, and sequencing analysis was performed. Monoclonal cell lines were screened according to the results. The primers for cloning screened were listed as Table 2.

**Table 2:** The oligonucleotide sequences of cloning screened primers.

Gene name	Primer sequences 5′‐3′
JAK2#2	F: ATCATTGCCTTCTTACATGCTTT
	R: TCTAGACCACCAAAACCAAAGT
JAK2#3	F: GAAATAACTGGCAGGAATACATCA
	R: CTAACATCTAACACAAGGTTGGC
SRC#2	F: GGGCCTCGTTTTCCCTATCTG
	R: GTCTTTTGCGCTTGCTCCACC
SRC#5	F: CCCAGCCTGGACATTCTTAATA
	R: TTTGGCTCTACTGCATTCCA

*F* forward, *R* reverse.

### PDX models

The study was approved by the Research Ethics Committee of Zhengzhou University. Five-to six-week-old SCID/CB17 female mice were purchased from Beijing Vital River Laboratory Animal Technology (Beijing, China). The tissues were cut into pieces of 8–10 mm^3^ and inoculated under the skin of the mice. Mice were randomly divided into three groups of LSG85 (*n* = 8) and HSG288 (*n* = 9) as follows: (1) vehicle; (2) vortioxetine hydrobromide (1.5 mg/kg); (3) vortioxetine hydrobromide (12 mg/kg). Mice were given vortioxetine hydrobromide or vehicle by gavage every day. When the tumor volume reached 1000 mm^3^, the mice were anesthetized and tumor tissues were excised. Tumor volume was calculated as follows: tumor volume (mm^3^) = (length × width^2^)/2.

### Immunohistochemical analysis

Formalin-fixed tumor tissues were paraffin embedded, cut into 4-μm sections and then placed on slides. The paraffin tissue sections were incubated at 65 °C for 2 h, deparaffinized, rehydrated, and processed for antigen retrieval. Next, following by incubation with 3% H_2_O_2_ for 10 min to inactivate endogenous peroxidase, then the tissues were incubated overnight with the Ki67 and p-STAT3 Y705 primary antibody (1:50) at 4 °C. The next day, all tissue sections were washed three times, and then incubated with the secondary antibody followed by 3, 3′-diaminobenzidine (DAB) staining. Then the sections were counterstained with hematoxylin, dehydration, and mounted with neutral resin. The Tissue Faxes (Tissue Gnostics) and Image Pro Plus software program (Media Cybernetics, Rockville, MD) were utilized to scan all sections and calculate positive cells.

### Statistical analysis

The Statistical Product and Service Solutions (SPSS 21.0, IBM, Inc. Armonk, NY, USA.) was used for all statistical analyses. Three independent experiments were conducted in cellular studies. The significance of differences was calculated by one-way ANOVA or non-parametric test. Quantitative data were shown as the mean values ± SD. *p* < 0.05 was considered statistically significant.

## Supplementary information

supplementary material

## Data Availability

The datasets analyzed during the current study are available in figshare (Dataset. 10.6084/m9.figshare.21586332) [45].
