# Supplementary material for: Vortioxetine hydrobromide inhibits the growth of gastric cancer cells in vivo and in vitro by targeting JAK2 and SRC
Source: Oncogenesis. 2023 May 5;12(1):24. doi: 10.1038/s41389-023-00472-4 (PMC10163056; doi:10.1038/s41389-023-00472-4)

Vortioxetine hydrobromide inhibits the growth of gastric cancer cells *in vivo* and *in vitro* by targeting JAK2 and SRC

Mingzhu Li ^1, 2*^, Lina Duan^1, 2*^, Wenjie Wu^1, 2^, Wenjing Li^1, 2^, Lili Zhao^1^, Ang Li^1, 2^, Xuebo Lu^1, 2^, Xinyu He^1, 2^, Zigang Dong^1, 2✉^, Kangdong Liu^1, 2, 3, 4, 5, 6✉^and Yanan Jiang^1, 2, 3✉^.

1 Department of Pathophysiology, School of Basic Medical Sciences, Academy of Medical Science, Zhengzhou University, Zhengzhou 450000, China

2 China-US (Henan) Hormel Cancer Institute, Zhengzhou 450000, Henan, China

3 State Key Laboratory of Esophageal Cancer Prevention and Treatment, Zhengzhou 450000, Henan, China

4 Provincial Cooperative Innovation Center for Cancer Chemoprevention, Zhengzhou University, Zhengzhou 450000, Henan, China

5 Cancer chemoprevention international collaboration laboratory, Zhengzhou 450000, Henan, China

6 Center for Basic Medical Research, Zhengzhou University, Zhengzhou 450000, Henan, China

*These authors contributed equally.

✉Corresponding authors: dongzg@zzu.edu.cn; kdliu@zzu.edu.cn; yananjiang@zzu.edu.cn.

**Supplementary material**

**Supplementary legends**

**Figure S1** Vortioxetine hydrobromide inhibited GC cell anchorage-independent and anchorage-dependent growth (Scale bar: 50 μm). **A** The pictures of soft agar assay after vortioxetine hydrobromide treatment. **B** The pictures of colony formation assay after vortioxetine hydrobromide treatment.

**Figure S2** Vortioxetine hydrobromide bound with JAK2 and SRC in intact cells. **A** The HGC27 cell lysates were incubated with vortioxetine hydrobromide-conjugated Sepharose 4B beads or with Sepharose 4B beads alone. The results were indicated by Western blotting. **B** The IC50 of vortioxetine hydrobromide with Src kinase or JAK2 kinase was assessed by Kinase-Lumi™ luminescent kinase assay. **C** The binding capacity of vortioxetine hydrobromide to JAK2 and SRC in AGS cells was determined by Western blotting. **D** The binding capacity of vortioxetine hydrobromide to JAK2 and SRC in HGC27 intact cells was determined by Western blotting.

**Figure S3** Vortioxetine hydrobromide inhibited STAT3 phosphorylation and nuclear translocation activity. **A** The pictures of Immunofluorescence staining of GC cells (Scale bar: 20 μm). **B** The nucleus localization changes of STAT3 after vortioxetine hydrobromide treatment in AGS cells. **C** The change of STAT3-mediated molecules after vortioxetine hydrobromide treatment in AGS cells. Mean ± S.D. (n=3) (* p < 0.05, ** p < 0.01, *** p < 0.001)

**Figure S4** JAK2 and SRC were up-regulated in GC and were positively correlated with poor clinical grade. **A**, **B** The mRNA levels of JAK2 and SRC in a variety of cancers based on the TCGA and GTEX database. **C**, **D** The mRNA levels of JAK2 in GC (TCGA and GTEx database) based on tumor histology (C) and clinical stages (D). **E**, **F** The mRNA levels of SRC in GC (TCGA and GTEX database) based on tumor histology (E) and clinical stages (F). Mean ± S.D. (* p < 0.05, ** p < 0.01, *** p < 0.001)

**Figure S5** Knockout of JAK2 or SRC reduced GC cells sensitivity to vortioxetine hydrobromide. **A** The pictures of sgJAK2 and sgSRC cells of colony formation assay. **B** The pictures of sgJAK2 and sgSRC cells in colony formation assay with various concentrations of vortioxetine hydrobromide treatment (0, 0.5, 1, 2, 4 μM).

**Figure S6** The sensitivity to vortioxetine hydrobromide was attenuated in JAK2 or SRC knockout monoclonal GC cells. **A** Knockout of JAK2 or SRC monoclonal in GC cells were assessed by Western blotting. **B** Cell viability after JAK2 or SRC knockout was assessed by MTT assay. **C** Colony numbers of JAK2 or SRC knockout monoclonal cells were measured by colony formation assay. **D** The inhibitory effect of vortioxetine hydrobromide on JAK2 or SRC knockout monoclonal cells was detected by proliferation assay after 96 h. Cell viability was evaluated by MTT assay and normalized to that of the control. **E** JAK2 or SRC knockout monoclonal cells were plated into 6-well plates and treated with 4 μM vortioxetine hydrobromide for 10 days, followed by crystal violet staining to monitor colony formation. Mean ± S.D. (n=3) (* p < 0.05, ** p < 0.01, *** p < 0.001)

**Figure S7** Knockout of JAK2 and SRC reduced GC cells sensitivity to vortioxetine hydrobromide. **A** The pictures of JAK2 and SRC knockout cells of colony formation assay. **B** The pictures of JAK2 and SRC dual knockout cells of colony formation assay with 4 μM vortioxetine hydrobromide treatment.

**Figure S8** Vortioxetine hydrobromide inhibited the growth of GC patient-derived xenograft (PDX). **A** Tumor sizes of individual mice in LSG85 (n=8) and HSG288 (n=9) cases. **B** The pictures of immunohistochemistry. **C** The pictures of HE staining in the tumor tissues (heart, liver, spleen, lung, kidney and brain) of mice. **D** Mice weights were measured every 2 days. (Scale bar: 50 μm) Mean ± S.D. (* p < 0.05, ** p < 0.01, *** p < 0.001)

**Figure S1**

**
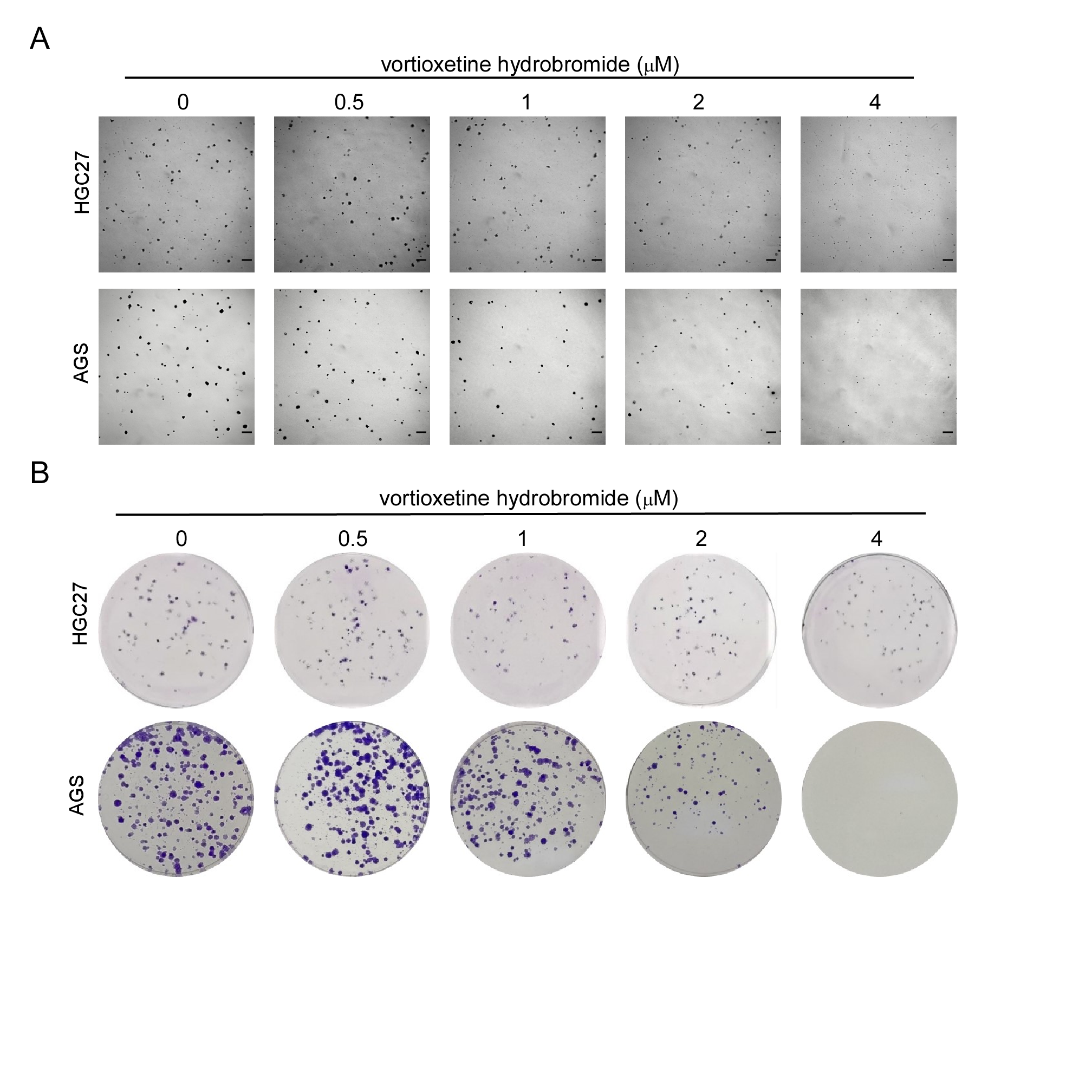
**

**Figure S2**

**
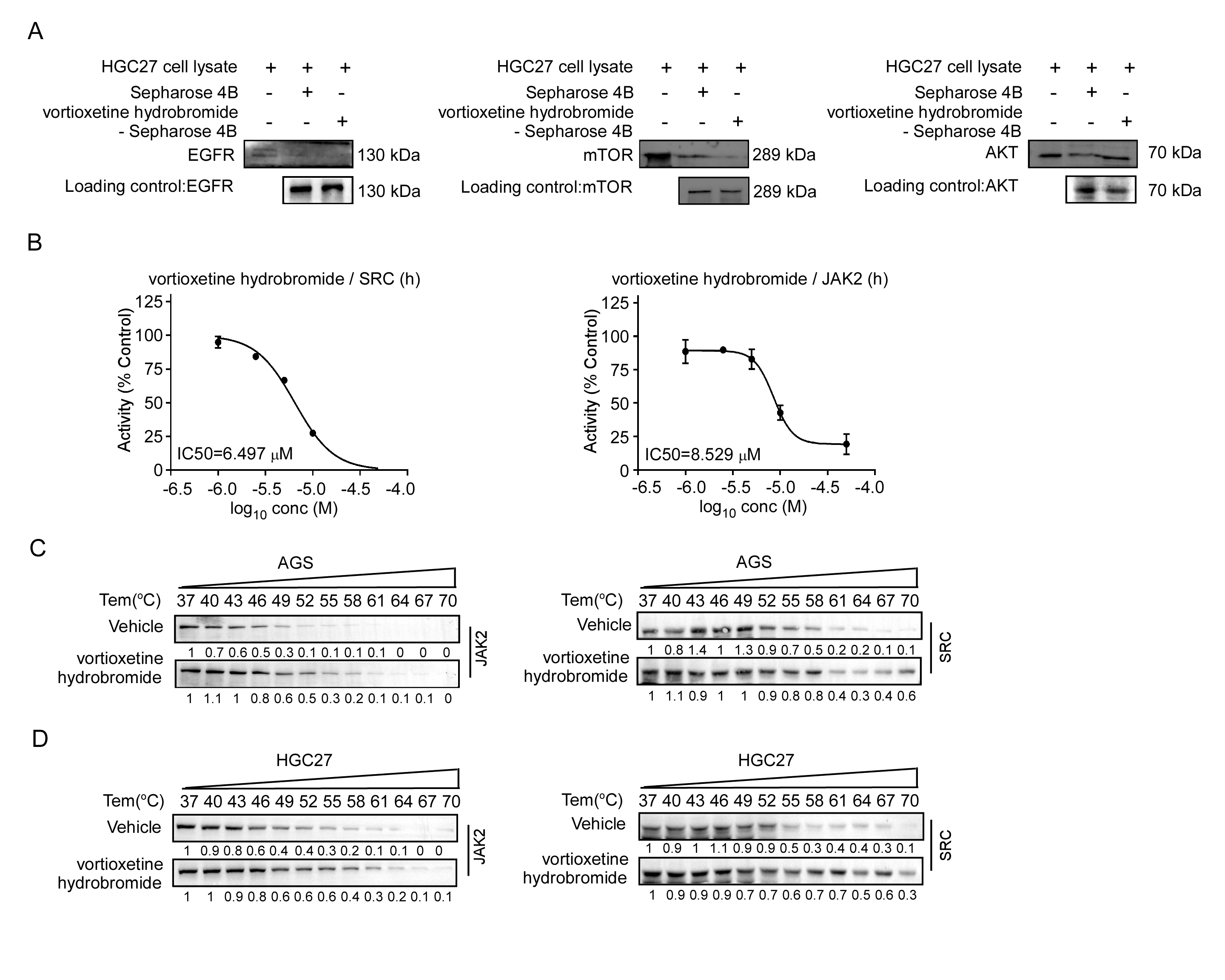
**

**Figure S3**

**Figure S4**

**
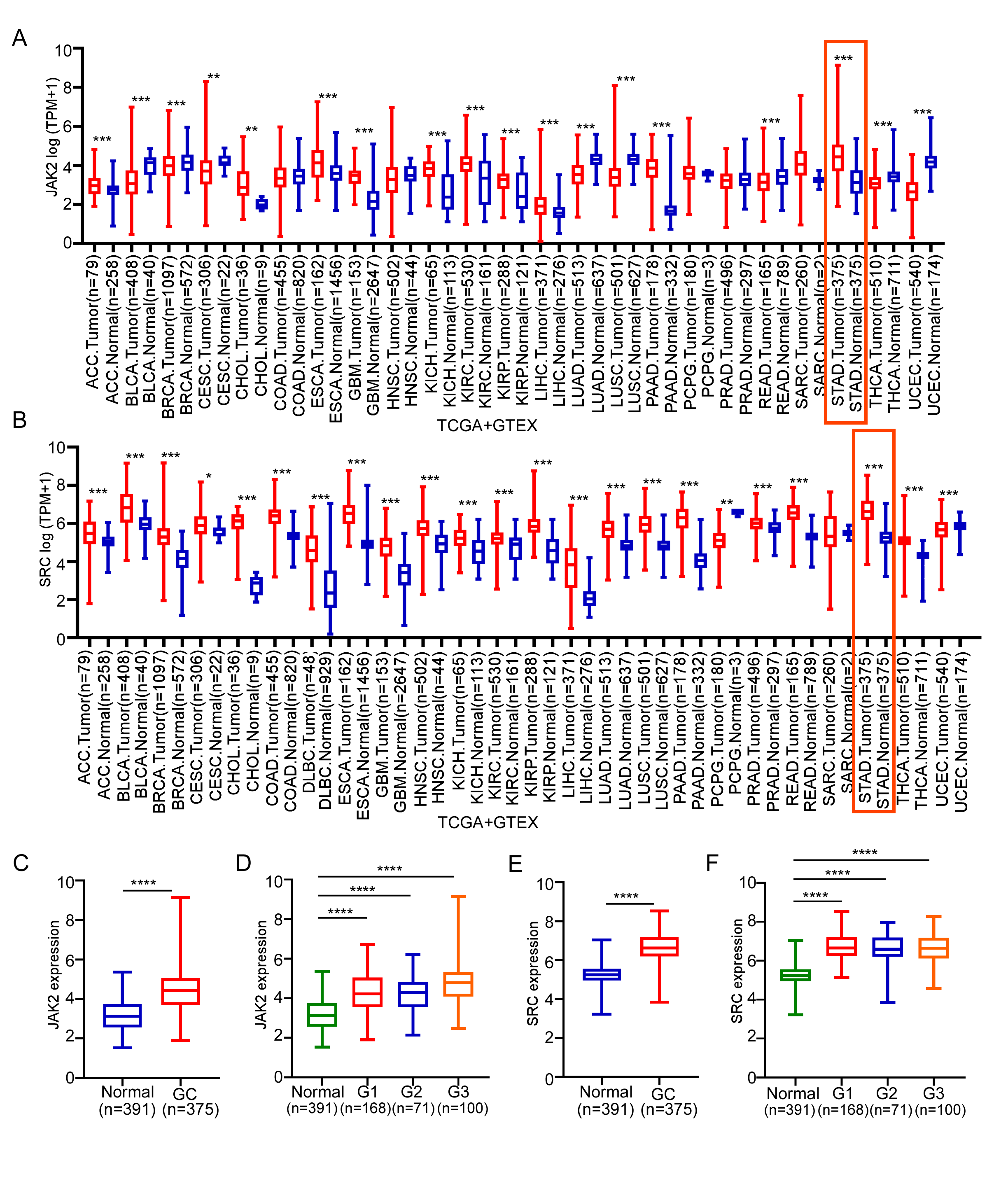
**

**Figure S5**


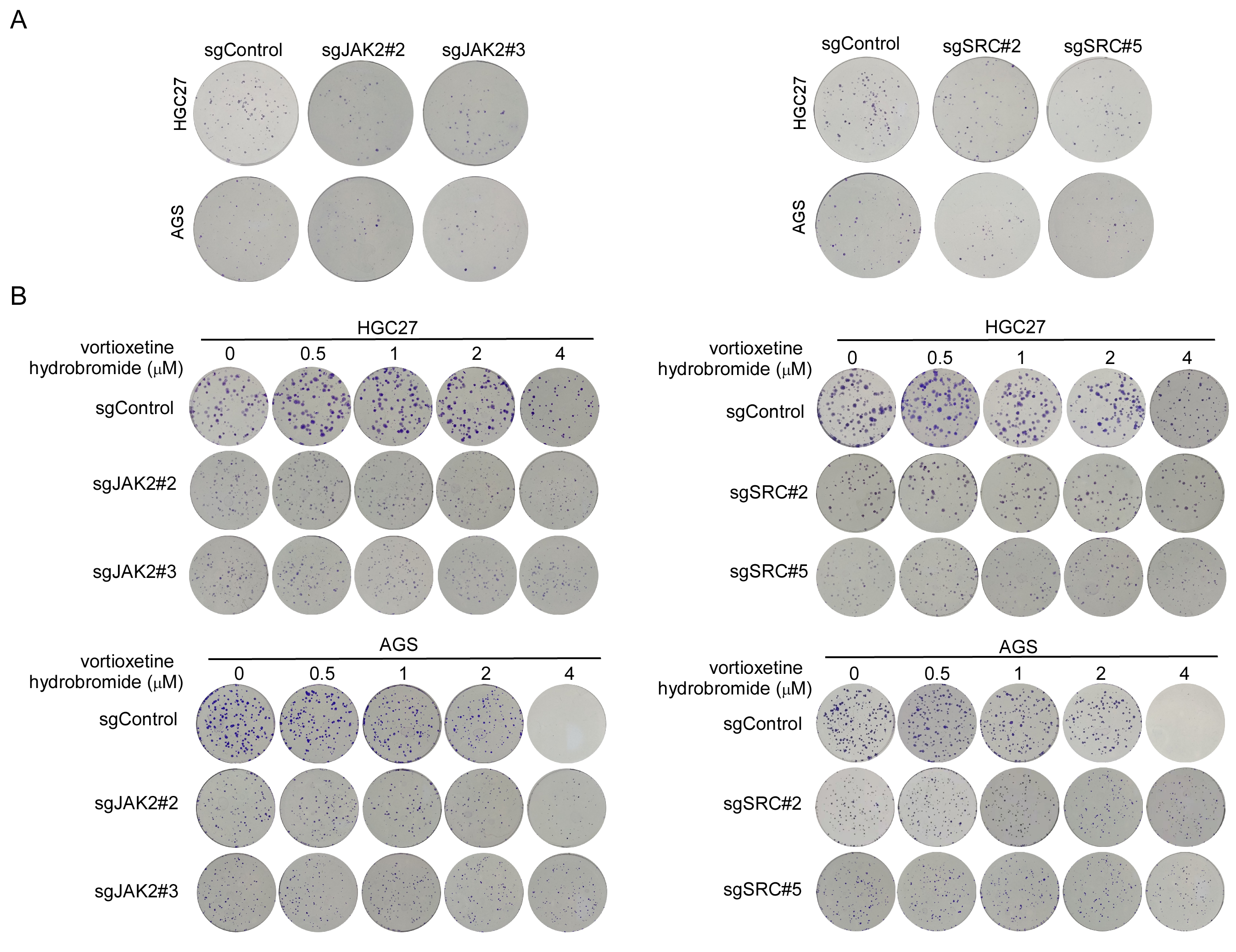


**
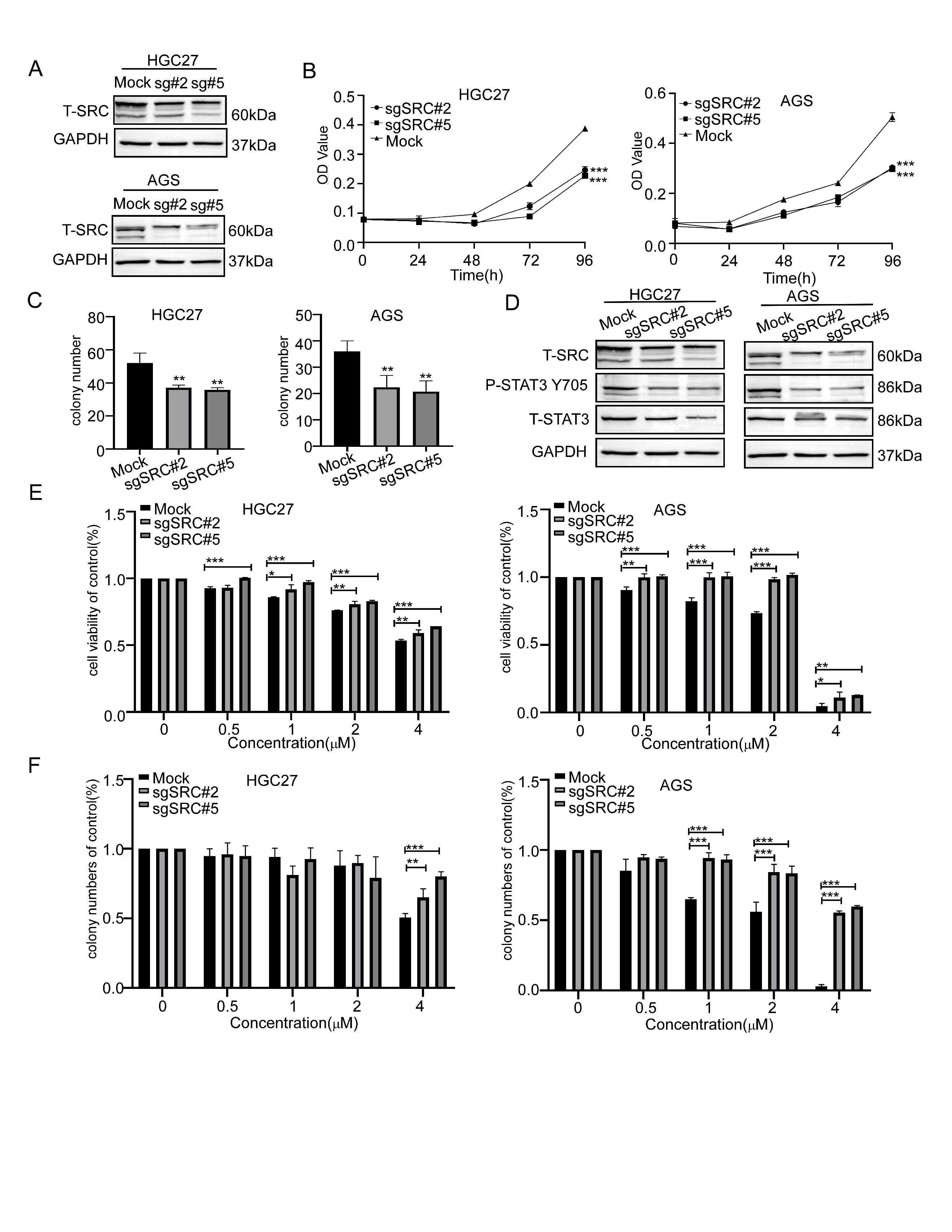
 Figure S6**

**FigureS7**

**
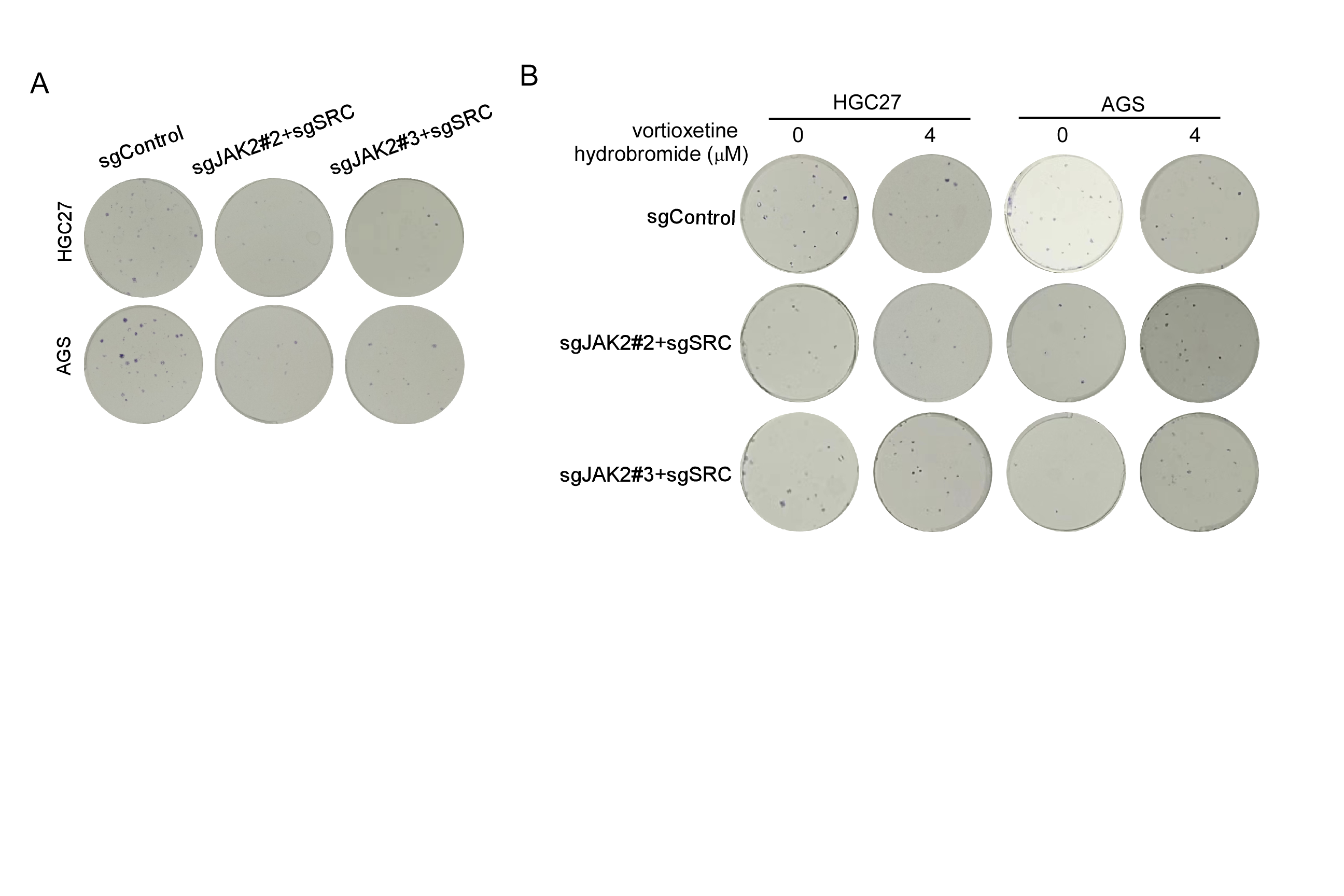
**

**Figure S8**


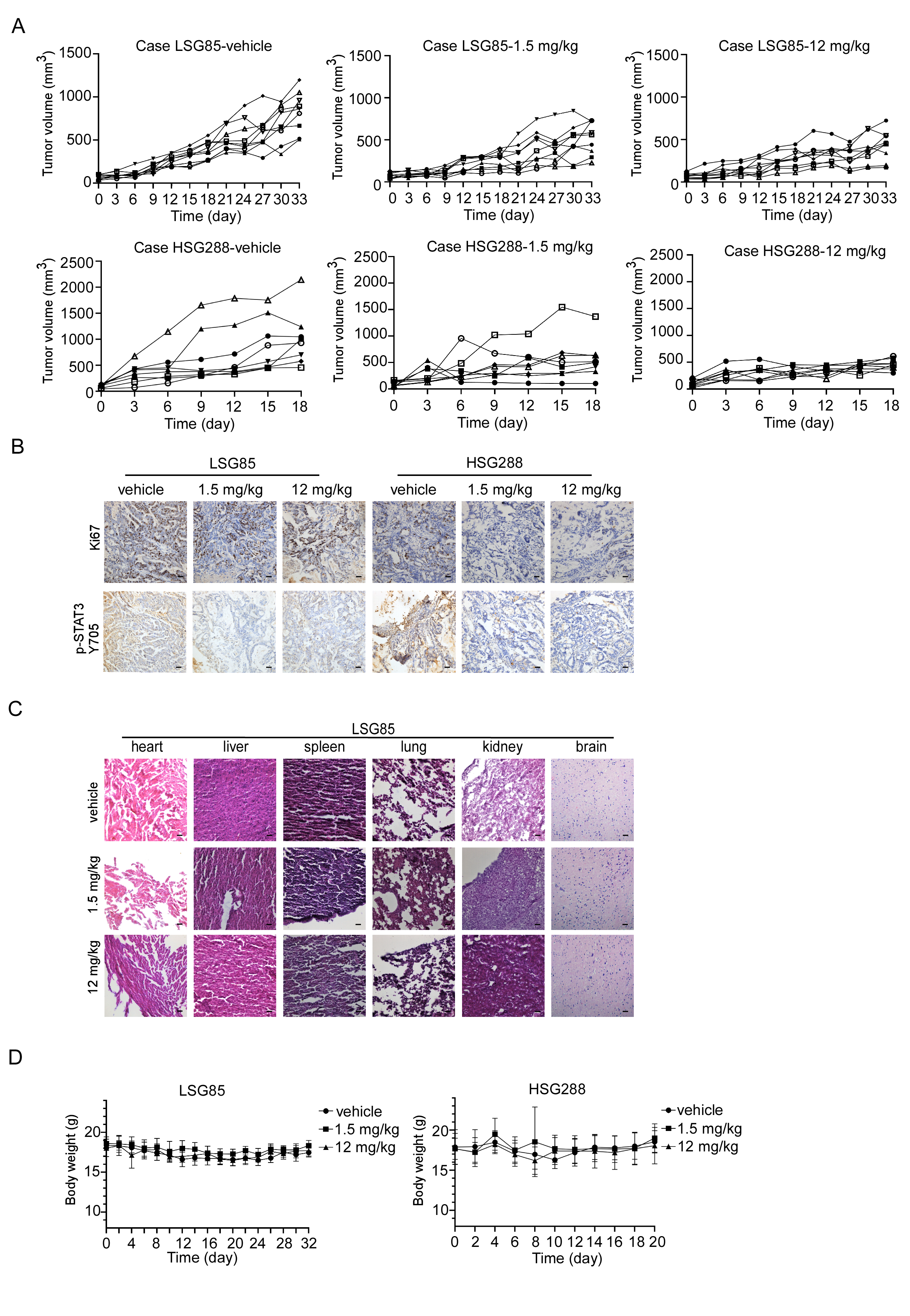

Supplement: Supplementary file 1 — supplementary material [file 41389_2023_472_MOESM1_ESM.docx]
